# IGS Minisatellites Useful for Race Differentiation in *Colletotrichum lentis* and a Likely Site of Small RNA Synthesis Affecting Pathogenicity

**DOI:** 10.1371/journal.pone.0137398

**Published:** 2015-09-04

**Authors:** Jonathan Durkin, John Bissett, Mohammadhadi Pahlavani, Brent Mooney, Lone Buchwaldt

**Affiliations:** 1 Agriculture and Agri-Food Canada, Saskatoon Research Centre, Saskatoon, Canada; 2 Agriculture and Agri-Food Canada, Eastern Cereal and Oilseed Research Centre, Ottawa, Canada; 3 Department of Agronomy and Plant Breeding, Gorgan University of Agricultural Sciences, Gorgan, Iran; Georg-August-University Goettingen, GERMANY

## Abstract

*Colletotrichum lentis* is a fungal pathogen of lentil in Canada but rarely reported elsewhere. Two races, Ct0 and Ct1, have been identified using differential lines. Our objective was to develop a PCR-probe differentiating these races. Sequences of the translation elongation factor 1α (*tef1α*), RNA polymerase II subunit B2 (*rpb2*), ATP citrate lyase subunit A (*acla*), and internal transcribed spacer (ITS) regions were monomorphic, while the intergenic spacer (IGS) region showed length polymorphisms at two minisatellites of 23 and 39 nucleotides (nt). A PCR-probe (39F/R) amplifying the 39 nt minisatellite was developed which subsequently revealed 1–5 minisatellites with 1–12 repeats in *C*. *lentis*. The probe differentiated race Ct1 isolates having 7, 9 or 7+9 repeats from race Ct0 having primarily 2 or 4 repeats, occasionally 5, 6, or 8, but never 7 or 9 repeats. These isolates were collected between 1991 and 1999. In a 2012 survey isolates with 2 and 4 repeats increased from 34% to 67%, while isolated with 7 or 9 repeats decreased from 40 to 4%, likely because Ct1 resistant lentil varieties had been grown. The 39 nt repeat was identified in *C*. *gloeosporioides*, *C*. *trifolii*, *Ascochyta lentis*, *Sclerotinia sclerotiorum* and *Botrytis cinerea*. Thus, the 39F/R PCR probe is not species specific, but can differentiate isolates based on repeat number. The 23 nt minisatellite in *C*. *lentis* exists as three length variants with ten sequence variations differentiating race Ct0 having 14 or 19 repeats from race Ct1 having 17 repeats, except for one isolate. RNA-translation of 23 nt repeats forms hairpins and has the appropriate length to suggest that IGS could be a site of small RNA synthesis, a hypothesis that warrants further investigation. Small RNA from fungal plant pathogens able to silence genes either in the host or pathogen thereby aiding infection have been reported.

## Introduction

Lentil (*Lens culinaris* subsp. *culinaris* Medic) is an important pulse crop for farmers in Western Canada both economically and as a beneficial rotational crop contributing to soil health and nitrogen fixation. Canada has been a leading producer of lentil since 2008 with an annual average production of 1.5 million tonnes of seed destined for human consumption and primarily exported to Bangladesh, Turkey, India and Egypt [FAOSTAT]. One of the major limitations to lentil production in Canada is the fungal disease, anthracnose, caused by the pathogen *Colletotrichum lentis* Damm, (synonym *C*. *truncatum* (Schwein.) Andrus & W.D. Moore [1]). The pathogen cause substantial defoliation and sever lesions on stems resulting in large patches of dying lentil plants in the field. Yield losses may reach 60% or higher if not controlled with fungicide application [2]. Lentil anthracnose has occasionally been reported in other countries including USA [3], Bulgaria, [4], Bangladesh, Ethiopia and Syria [5].

Morphological characteristics and host range combined with molecular analysis of coding and non-coding genomic regions have been used to define fungal species and their phylogenetic relationships. Species in the *Colletotrichum* genus have been difficult to characterize and is still a matter of debate [6]. *Colletotrichum* isolates obtained from lentil in Canada were originally classified as *C*. *truncatum*, however for the purpose of this paper these will be designated *C*. *lentis* as recently described by Damm et al. [7]. Pathogenic variability of *C*. *lentis* collected from lentil between 1991 and 1999 in the Canadian Provinces of Manitoba and Saskatchewan was examined by Buchwaldt et al. [8]. This study differentiated two races, Ct0 and Ct1, based on inoculation of differential lentil lines. Isolates belonging to race Ct0 were pathogenic on the variety Indianhead and a number of germplasm lines, while isolates belonging to race Ct1 were non-pathogenic on these lines. Screening of 50 *C*. *lentis* single spore isolates showed that the two races were equally frequent [8]. At that time, all commercial lentil varieties were susceptible to both races, thus there was no selection pressure on the pathogen population. Since then several lentil varieties were developed with resistance to race Ct1 derived from cv. Indianhead [9, 10], and about one third of current varieties have some resistance to this race [Saskatchewan Ministry of Agriculture 2014].

DNA polymorphisms in the internal transcribed spacer (ITS) regions and the intergenic spacer (IGS) and of ribosomal DNA (rDNA) either alone or in combination with various protein-coding regions have been used to differentiate *Colletotrichum* at the species level that in general support groupings based on host range and pathogen morphology [6]. Nuclear rDNA in fungi exists as a series of genes extensively duplicated at a single locus named the nuclear organizing region (NOR) packaged with the help of specific proteins to form the nucleolus. Each rDNA unit is linearly organized in ribosome-coding genes and non-coding, single stranded regions as follows, 26S (large ribosomal unit), ITS1, 5.8S, ITS2, IGS, and 18S (small ribosomal unit). One additional ribosomal gene, 5S, occurs either once in every IGS region or as multiple copies dispersed throughout the genome. The ribosomal encoding genes have a low rate of mutation and have not functionally changed since the separation of fungi from its eukaryotic ancestors. Thus, these genes are suitable for determining the evolutionary relationships between fungi at the phylum to order levels. The ITS and IGS regions have faster mutation rates resulting in molecular variations that have proven suitable for discernment of closely related fungal species and occasionally intra-species differences at the isolate level. Furthermore, the conserved sequences of the ribosomal genes permit design of standard primers anchored in 18S and 26S coding regions for amplification into the non-coding ITS and IGS regions.

Ribosomal genes are extensively duplicated in the genome allowing organisms to rapidly increase production of ribosomes during growth or in response to external stimuli. On the technical level, this is an advantage since PCR amplification can be undertaken from small amount of initial DNA. Our initial objective was to develop a PCR-based probe to differentiate races of *C*. *lentis*. Concurrently, a minisatellite was discovered in the IGS resembling a site of small RNA synthesis which we hypothesize could explain differences in pathogenicity of the races.

## Results

### Pathogenicity of *C*. *lentis* races

A total of 50 *C*. *lentis* isolates were collected in lentil fields between 1991 and 1999. Isolates were characterized for pathogenicity by inoculation of lentil differentials and comprised 26 race Ct0 and 24 race Ct1 some of which were published previously [8]. The two groups of isolates were the basis for comparative DNA sequence analysis and development of a PCR-based race-specific probe (Table 1).

**Table 1:** Differentiation of two *Colletotrichum lentis* races based on repeat variations of a 39 nt minisatellite in IGS. The IGS, ITS and three genes, *tef1α*, *rpb2* and *acla* were sequenced in isolates representative of the two races. A probe, 39F/R, amplified 7 and 9 of the 39 nt repeats in race Ct1 isolates, while race Ct0 isolates had from 2 to 12 repeats, but never 7 or 9. The number of the 39 nt repeat was determined by gel electrophoresis and verified by sequencing of DNA extracted from gel bands.

Isolate	Year, Province	Sequenced region	NCBI accession	IGS sequence	Major band	Minor band	DNA sequence of band
Race Ct0 isolates							
95A8*	1995, MB	IGS, *tef-1α*	IGS, KM818497	4	4		4
95D35*	1995, MB				4		
95H15*	1995, MB	IGS, *tef-1α*, *rpb2*, *acla*	IGS, KM818504	4	4		4
95N8*	1995, MB	IGS, ITS, *tef-1α*	IGS, KM818500	4	4		4
95N15*	1995, MB				2		
92R4*	1992, MB	IGS, ITS, *tef-1α*	IGS, KM818501	4	4		4
95S27*	1995, MB	IGS, ITS, *tef-1α*	IGS, KM818498	4	4		4
95SP31*	1995, MB	IGS, ITS, *tef-1α*, *acla*	IGS, KM818499	4	4		4
Apr9920*	1999, SK				4		
Apr9926	1999, SK				4		
May9902	1999, SK				4		
May9908*	1999, SK				4		
May9915	1999, SK				4		
May9917	1999, SK				4		
May9922	1999, SK				4		
Jun9903*	1999, SK				4		
95A10* #	1995, MB				8	2	8
Apr9906	1999, SK				2	12	
Apr9908*	1999, SK				2	12	2
Apr9921*	1999, SK				2	12	
May9941#	1999, SK				5	12	5, 12
May9918#	1999, SK				5, 6	12	
95D1 *	1995, MB	*rpb2*, *acla*			2	6, 12	2, 6, 12
95N10*	1995, MB	IGS,	IGS, KM818502	2	2	3, 5, 12	2
95N1*	1995, MB	IGS, *tef-1α*	IGS, KM818503	2	2	5, 6, 12	2
May9914	1999, SK				2	4, 5, 6, 12	
Race Ct1 isolates							
1a*	1999,SK				7		7
95N2* #	1995, MB	IGS, ITS, *tef-1α*	IGS, KM818508	7	7		7
95N11*	1995, MB	IGS, *rpb2*, *acla*	IGS, KM818507	7	7		7
95N20*	1995, MB	IGS, ITS, *tef-1α*	IGS, KM818509	7	7		7
95N21*	1995, MB		IGS, KM818506	7	7		7
Apr9903*	1999, SK				7		
Apr9907	1999, SK				7		
Apr9909	1999, SK				7		
Apr9919	1999, SK				7		
May9913	1999, SK				7		
Jul9901	1999, SK				7		
Jul9902	1999, SK				7		
95B36*	1995, MB	IGS, ITS, *tef-1α*	IGS, KM818511	9	9		9
91Chl*	1991, SK	IGS, ITS, *tef-1α*, *rpb2*, *acla*	IGS, KM818512	7	7, 9		7
95N23*	1995, MB	IGS, *rpb2*, *acla*			7, 9		
72126	1999, SK				7, 9		
Apr9924	1999, SK				7, 9		
May9919	1999, SK				7, 9		
May9939	1999, SK				7, 9		
91B11*	1991, MB	IGS, ITS, *tef-1α*	IGS, KM818505	7	7	9	7, 9
95S12*	1995, MB	IGS, ITS, *tef-1α*	IGS, KM818510	7	7	9	7
Jun9902	1999, SK				7	9	
95N12*	1995, MB				9	7	
Apr9910	1999, SK				9	7	

* Isolates published by Buchwaldt et al. (2004).

^#^ Used for production of additional single spore isolates.

SK = Saskatchewan, MB = Manitoba. *tef1α* = translation elongation factor 1α, *rpb2* = RNA polymerase II subunit B2, *acla* = ATP citrate lyase subunit A.

### Monomorphism in ITS, *tef1α*, *rpb2* and *acla*

**Fig 1:**
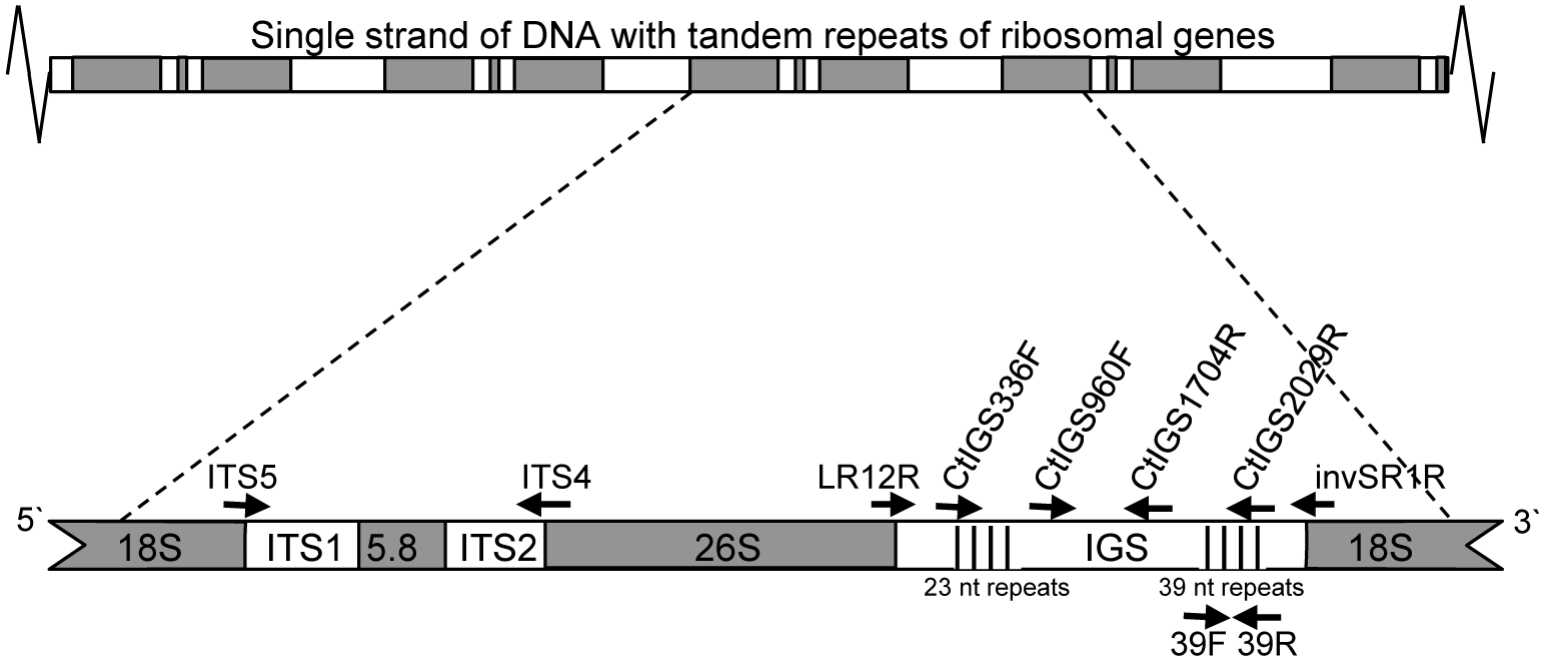
Organisation of ribosomal DNA in *Colletotrichum lentis* and the location of primers. Ribosomal genes (18S, 5.8S and 26S), internal transcribed and intergenic spacer non-coding regions (ITS and IGS). Two minisatellites of 23 and 39 nucleotides are shown. Primer pair 39F and 39R amplified a length polymorphism differentiating two races of *Colletotrichum lentis* from lentil.

The internal transcribed spacers, ITS1 and ITS2, of rDNA (Fig 1) and three genic regions were amplified and sequenced using generic primers (S1 Table) in 16 *C*. *lentis* isolates from lentil representing the two races, Ct0 and Ct1 (Table 1). This allowed alignment and comparison of 609 nucleotides (nt) in ITS, 587 nt in *tef1α*, 944 nt in *rpb2*, and 894 nt in *acla* spanning seven intron-exon regions. Surprisingly, no polymorphisms of any kind could be identified at these loci. Since the DNA sequences were monomorphic for all isolates a single sequence at each locus was submitted to the National Center for Biotechnology Information (NCBI) for isolate Ct0 95SP31. ITS (KM818516), *tef1a* (KM818513), *rpb2* (KM818514) and *acla* (KM818515).

### Polymorphisms in IGS

**Fig 2:**
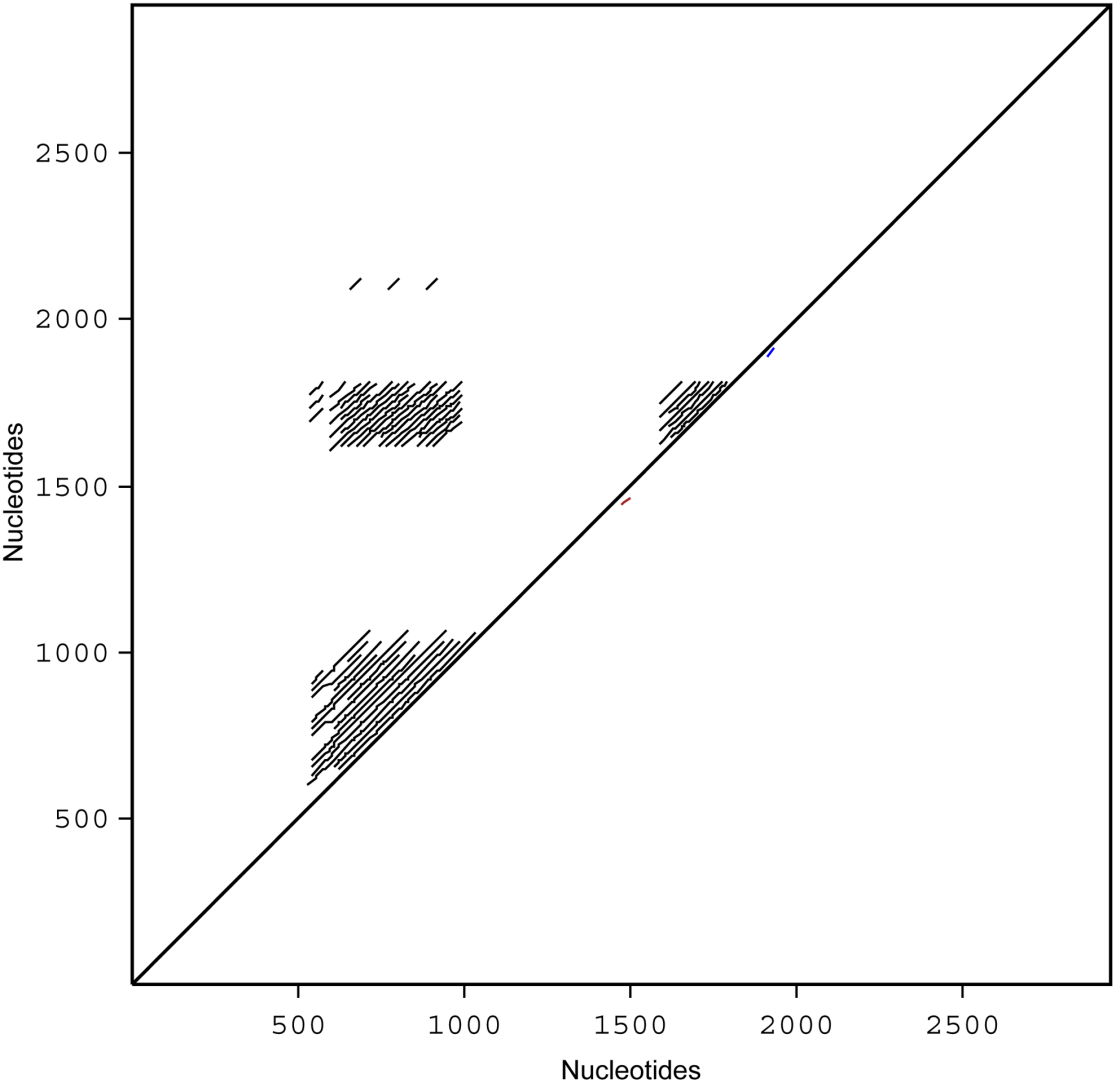
Dot matrix representing sequence similarities in IGS of *Colletotrichum lentis*. A 23 nt minisatellite was identified 550–1000 nucleotides downstream of the 18S gene, and another 39 nt minisatellite was identified at 1600–1880 nucleotides. Sequence similarity between the two minisatellites is evident where these areas intersect.

**Fig 3:**
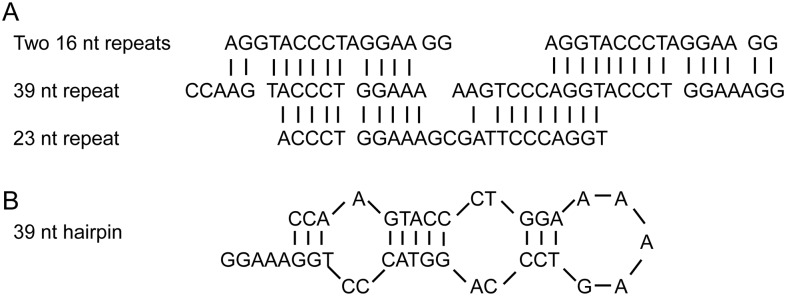
Alignment of sequence repeats in IGS of *Colletotrichum lentis* and secondary folding of a 39 nt minisatellite. A) Sequence similarity between the 16 nt pre-repeat, the 39 nt repeat and the 23 nt repeat (repeat F, Table 3). B) Secondary folding of a single 39 nt repeat into a stable hairpin structure.

The IGS region was initially amplified in three Ct0 and four Ct1 isolates using generic primers, LR12R and invSR1 [Vilgalys’ laboratory], annealing to the 5’- end of 26S and the 3’- end of 18S ribosomal genes, respectively (Fig 1). Four *C*. *lentis*-specific primers, CtIGS336F, CtIGS960F, CtIGS1704R and CtIGS2029R, were subsequently designed which allowed amplification and sequencing of the complete IGS region in eight Ct0 and eight Ct1 isolates (Fig 1). Alignment of the resulting DNA sequences revealed that the 5S gene was absent, thus IGS comprise a single region in *C*. *lentis*. Two polymorphic minisatellites were identified, the first starting 545 nt downstream of the 26S gene containing a varying number of 23 nt imperfect repeats, and the second starting 1658 nt downstream consisting of a varying number of 39 nt perfect repeats (Fig 1). A dot matrix plot of the complete IGS sequence from a single representative isolate (Ct0 95SP31) showed three areas of sequence similarities (Fig 2). The 23 nt and 39 nt minisatellites were identified from 550 nt to 1000 nt and from 1600 nt to 1880 nt, respectively. A third area was evident where the two first areas intersected thereby demonstrating sequence similarities between the 23 nt and 39 nt minisatellites. The sequence similarity between the 39 nt repeat and each of ten different 23 nt repeats ranged from 60 to 78% (Fig 3A). Other similarities included a 16 nt pre-repeat in the 39 nt minisatellite, which aligned with both the 5’- end and the 3’- end of the 39 nt repeat as shown in Fig 3A.

Ten of the nucleotides in the 23 nt minisatellite were conserved, ACCCxxGxxxxxxxTTCCxxxGx, while the remaining varied slightly in ten patterns, A to J (Table 2). Three length variations were present among the 18 *C*. *lentis* isolates consisting of 14, 17 or 19 repeats, which theoretically would allow an infinite number of nucleotide variations. However, only a single nucleotide sequence existed for each of the three length variations (Table 3). All Ct1 isolates had 17 repeats, while Ct0 isolates had either 14 or 19 repeats, except Ct0 isolate 95H15 which had the same sequence as Ct1 isolates. The 23 nt minisatellite was not used for development of a race specific PCRprobe primarily because sequencing of the entire region would be necessary to capture the genotypic variation in each isolate. Analysis of the secondary DNA folding of the three length variations resulted in several possible hairpin structures with low free energy values ranging from -42.3 to -55.7 kcal mol^-1^ (S2 Table).

**Table 2:** Differentiation of two *Colletotrichum lentis* races based on sequence variation of a 23 nt minisatellite in IGS. There were ten groups of sequence variations; A to J (conserved nt in bold). Isolates grouped according to race Ct0 and Ct1 except isolate Ct0 95H15. The corresponding RNA sequences have uracil in position 1.

Repeat name	23 nt DNA	23 nt RNA	Race Ct0 isolates	Race Ct1 isolates
A	**ACCC**TG**G**AGAGCGA**TTCC**CTA**G**T	**UGGG**AC**C**TCTCGCU**AAGG**GAU**C**A	All	All
B	**ACCC**TG**G**AGCAGAA**TTCC**CGA**G**T	**UGGG**AC**C**UCGUCUU**AAGG**GCU**C**A	All	All
C	**ACCC**CG**G**AAAGCGA**TTCC**CAA**G**T	**UGGG**GC**C**UUUCGCU**AAGG**GUU**C**A	All	All
D	**ACCC**TG**G**AAAGCAG**TTCC**CAA**G**T	**UGGG**AC**C**UUUGCUC**AAGG**GUU**C**A	All	All
E	**ACCC**TG**G**AGCAGAA**TTCC**CAG**G**T	**UGGG**AC**C**UCGUCUU**AAGG**GTC**C**A	All	All
F	**ACCC**CG**G**AAAGCGA**TTCC**CAG**G**T	**UGGG**GC**C**UUUCGCU**AAGG**GUC**C**A	None*	All
G	**ACCC**TG**G**AAAGCAA**TTCC**TAC**G**C	**UGGG**AC**C**UUUCGUU**AAGG**AUG**C**G	All	All
H	**ACCC**TG**G**AAAGCAA**TTCC**CAA**G**T	**UGGG**AC**C**UUUCGUU**AAGG**GUU**C**A	All*	None
I	**ACCC**TA**G**CGCGGAA**TTCC**CAG**G**T	**UGGG**AU**C**GCGCCUU**AAGG**GTC**C**A	All	All
J	**ACCC**TA**G**GGCGGAA**TTCC**CAA**G**T	**UGGG**AU**C**CCGCCUU**AAGG**GUU**C**A	All	All

**Table 3:** Differentiation of two *Colletotrichum lentis* races based on three repeat variations of a 23 nt minisatellite in IGS. Repeat A to J refer to DNA sequences in Table 2. Isolates grouped according to race Ct0 and Ct1 except isolate Ct0 95H15.

Number of 23 nt repeats	Repeat pattern in the 23 nt minisatellite	*C*. *truncatum* races and isolates
14	A_B,C,D,E,_________G,B,C,H,E,G,I,G,J	Race Ct0 95D1, 95N1, 95N10
17	A_B,C,B,F,G,B,C,D,E,G,B,C____,G,I,G,J	All race Ct1 and Ct0 95H15
19	A_B,C,D,E,G,B,C,D,E,G,B,C,H,E,G,I,G,J	Race Ct0 95A8, 95N8, 92R4, 95S27, 95SP31

The complete IGS sequence from 16 *C*. *lentis* isolates is available at NCBI. Isolate 95A8 (KM818497), 95S27 (KM818498), 95SP31 (KM818499), 95N8 (KM818500), 92R4 (KM818501), 95N10 (KM818502), 95N1 (KM818503) and 95H15 (9KM818504). Race Ct1 isolates: 91B11 (KM818505), 95N21 (KM818506), 95N11 (KM818507), 95N2 (KM818508), 95N20 (KM818509), 95S12 (KM818510), 95B36 (KM818511) and 91Chl (KM818512).

### Race specificity of the 39F/R PCR probe

**Fig 4:**
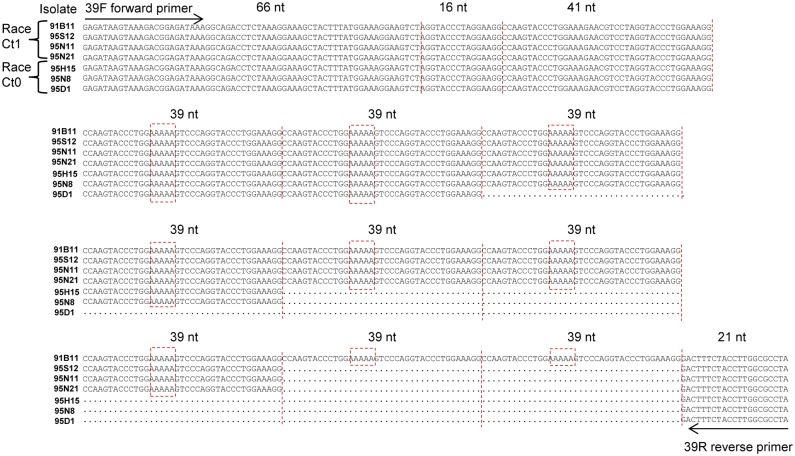
Alignment of a 39 nt minisatellite in IGS of *Colletotrichum lentis* isolates differentiating race Ct0 from Ct1 based on length polymorphisms. Arrows show the forward (39F) and reverse (39R) primers. Lines demarcate the 16 nt and 41 nt pre-repeats and each of the following 39 nt repeats. Boxes show five adenosine residues that form a hairpin loop in secondary folding (Fig 3B).

The 39 nt minisatellite contained a varying number of the following prefect repeat, CCA AGT ACC CTG GAA AAA GTC CCA GGT ACC CTG GAA AGG (Fig 4). Alignment showed a maximum length of 2726 nt in Ct0 isolates and 3089 nt in Ct1 isolates. In this initial group of isolates, Ct0 isolates consistently had 2 or 4 repeats, while Ct1 isolates had 7 or 9 repeats. This length polymorphism was therefore selected for development of a PCR-based probe to differentiate isolates in a subsequent field survey. A forward primer 39F (GAG ATA AGT AAA GAC GGA GAT AAA) and reverse primer 39R (TAG GCG CCA AGG TAG AAA GT) flanking the 39 nt minisatellite were designed. PCR amplification of length variants were separated by gel electrophoresis, stained and visualized under UV light. The 39F/R PCR probe was subsequently used to amplify the 39 nt minisatellite in 50 *C*. *lentis* isolates from lentil collected between 1991 and 1999 which had been characterized as either race Ct0 or Ct1 by inoculation of differential lentil lines (Table 1). Based on band size in agarose gels, results confirmed that race Ct1 isolates reliably had one or two minisatellites of either 7 or 9 (or both 7 and 9) of the 39 nt repeat, while Ct0 isolates had one to five minisatellites with primarily 2 or 4 repeats, occasionally 3, 5, 6 or 12, but never 7 or 9 repeats (Table 1). When DNA was sequenced from selected bands the presence of the 39 nt perfect repeat was evident in all cases regardless of band size and brightness.

**Fig 5:**
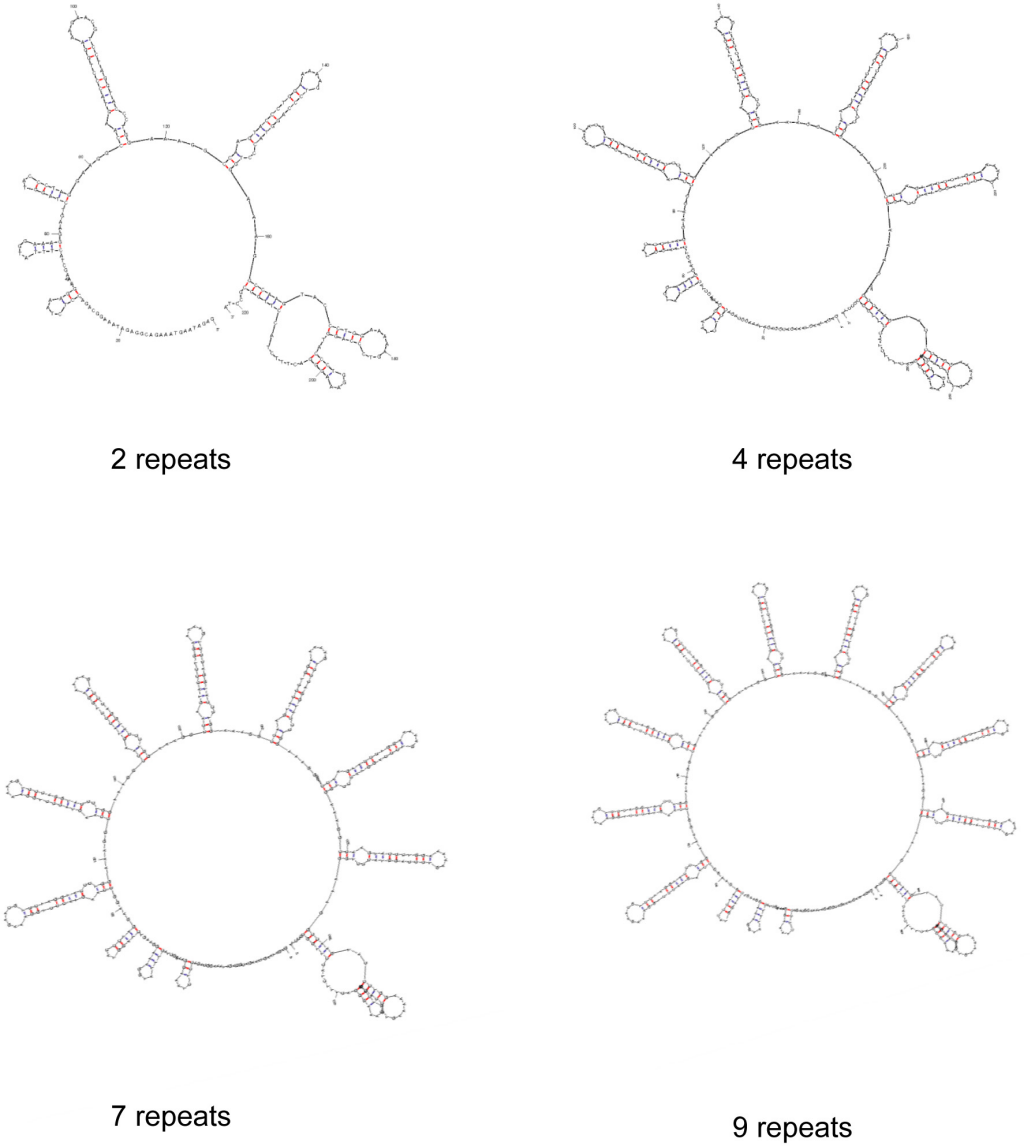
Predicted secondary structure of a 39 nt minisatellite in IGS of *Colletotrichum lentis*. Ct0 isolates most commonly have a minisatellites with 2 and 4 repeats of 39 nucleotides while Ct1 isolates have 7 and 9 repeats.

Analysis of secondary DNA folding of the 39 nt minisatellite resulted in stable stem-and-loop hairpin structures (Fig 5). Increasing the number of 39 nt repeats from two to twelve reduced the free energy value from -25.1 to -94.8 kcal mol^-1^ (S2 Table). Stepwise increase of repeats expanded the number of possible configurations from five to twenty. However, the free energy of different configurations with identical repeat length varied by only 2% (data not shown). The most energy efficient configuration consisted of two short hairpins formed by the 65 nt sequence containing the annealing site of the forward primer (39F), a short hairpin formed by the 16 nt pre-repeat, followed by a longer hairpin formed the 41 nt pre-repeat, and a hairpin for each of the following 39 nt repeats. The last 39 nt repeat together with the 21 nt containing the annealing site of the reverse primer (39R) formed a large loop with two small hairpins. The secondary DNA folding of the 39 nt minisatellite with 2 and 4 repeats common in Ct0 isolates and 7 and 9 repeats in Ct1 isolates are shown in Fig 5.

### Field survey of *C*. *lentis*

The two *C*. *lentis* races were identified at equal frequency among isolates collected between 1991 and 1999 in Western Canada as determined by both inoculation of differential lentil lines and the 39F/R PCR probe (Table 1). In 2012, the 39F/R probe was used to amplify the 39nt repeat in 219 *C*. *lentis* isolates from infected lentil seed samples collected across the Province of Saskatchewan (Table 4 and S3 Table). The frequency of repeats had changed since the first collection. Isolates with 2 or 4 repeats had increased from 33 to 67%, while isolates with 7 or 9 repeats decreased from 40 to 4%. If the probe accurately differentiates the two races then race Ct0 was more prevalent in the later survey.

**Table 4:** Frequency of a 39 nt repeat in IGS amplified by PCR probe 39F/R in 219 *Colletotrichum lentis* isolates collected in 2012. Amplification products were separated by gel electrophoresis and visualized by GelRed staining. The number of repeats was calculated as (band size– 144) / 39).

Number of isolates	Number of 39 nt repeats in IGS region	
	Major bands	Minor bands
18	2	
1	3	
132	4	
12	5	
1	6	
6	8	
2	2, 4	
1	3, 4	
1	3, 6	
1	6, 8	
1	2, 6	12
2	2	4
3	7	
7	9	
1	2	6
1	2	11
1	2	12
1	2	4, 5
1	2	4, 6
1	2	4, 12
1	2	5, 11
2	2	6, 12
1	2	5, 6
2	2	5, 6, 11
2	4	2
1	4	5
2	4	6
1	5	2
1	5	12
1	5	11, 12
1	5	12, 13
1	6	5
2	6	12
1	6	5, 11
1	8	3
1	8	12
1	11	5
1		2, 4, 11
2		5, 12
Total 219		

The 39F/R probe was also used to examine variations in the 39 nt minisatellite of 54 single spore isolates derived from each of four *C*. *lentis* isolates. All single spore isolates derived from race Ct0 isolate 95A10 had a single band corresponding to 8 repeats, isolates from May9918 had two bands equivalent to 5 and 6 repeats, and isolates from May9941 showed two bands of 5 and 12 repeats. Similarly, all single spore isolates derived from race Ct1 isolate 95N2 had a single band corresponding to 7 repeats. Clearly, the 39 nt minisatellite in all single spore isolates were identical to each of the original isolates. The banding patterns of some of these single spore isolates are shown in S2 Fig.

### 39 nt minisatellite in other fungal plant pathogens

The 39F/R PCR probe produced a wide range of amplification products in other fungal species. Sequencing of DNA extracted from selected bands showed the same 39 nt perfect repeat as in *C*. *lentis*, with the number of repeats corresponding to band sizes in gel electrophoresis. Isolates of *S*. *sclerotiorum* had 1–2 minisatellites with 2–6 repeats, *B*. *cinerea* 2–5 minisatellites with 2–7 repeats, *A*. *lentis* 2–6 minisatellites with 2–18 repeats, *C*. *gloeosporioides* 3–4 minisatellites with 2–37 repeats, *C*. *trifolii* 3–6 minisatellites with 2–40 repeats and the scentless chamomile biocontrol agent *C*. *truncatum* had 4 minisatellites with 2–40 repeats (Table 5 and S3 Fig). Evidently, the identical 39 nt minisatellite was present in all seven fungal species examined.

**Table 5:** Number of a 39 nt repeat in IGS amplified in seven fungal plant pathogenic species using rDNA probe 39F/R showing the universal presence of this minisatellite. The number of repeats was calculated as (band size– 144) / 39. The number of repeats was determined by gel electrophoresis and verified by sequencing of DNA extracted from gel bands.

			Number of 39nt repeats in IGS		
			Gel electrophoresis		
Host species	Fungal species	Isolate name	Major band	Minor band	DNA sequencing
Lentil	*S*. *sclerotiorum*	F0201	2		
		Jn9402	2	4	2
		Feb9808	2	6	
	*B*. *cinerea*	F9807	2	7	
		F0204	2	4, 5	
		Oct0401	2	3, 4, 5, 7	2
	*C*. *truncatum* Ct0	95A8	4		4
		92R4	4		4
		95N8	4		4
	*C*. *truncatum* Ct1	91B11	7	9	7, 9
		91Chl	7, 9		7
		95N2	7		7
	*A*. *lentis*	Nov9609	2	4	2
		Jan9902	2	4	
		MOO17	2	3, 4	
		Aug00A4	2	3, 4, ~14, ~18	2, 4
		Aug00C6	2, 4	3, 7, ~14, ~15	4
Soybean	*C*. *gloeosporioides*	6-7-1		2, 10, ~22	
		5-5-3	8, ~22	2, ~17	8
		2-4-1	~22	6, ~37	
Alfalfa	*C*. *trifolii*	1–07	2	4, 5	
		1–01	2	4, 5, 7, 9	2, 4, 5
		1–04	~40	2, 4, 7, 9, ~18	
Scentless chamomile	*C*. *truncatum* biocontrol agent	3B1	~40	2, 9, 11	

### Species specificity of the G02FP/G02RP PCR probe

Differentiation of *C*. *lentis* from other fungal species was possible using a previously published DNA probe, G02FP/G02RP [11], which amplified the expected 1600 nt DNA sequence only in *C*. *lentis* from lentil, whereas no amplicons were seen in any of the other six fungal species described above. As anticipated, the G02FP/G02RP PCR probe was unable to distinguish between race Ct0 and Ct1 of *C*. *lentis* in the current study.

## Discussion

No molecular polymorphisms were found among *C*. *lentis* isolates from lentil in ITS or seven exon-intron spanning regions of three genes, *tef1α*, *rpb2*, and *acla*, used in other phylogenetic studies [6, 12, 13]. Conversely, the IGS region was highly polymorphic encompassing two minisatellites with 23 nt and 39 nt repeats, respectively. The fact that the identical 39 nt repeat was conserved in all fungal species we examined provide evidence for its early evolutionary origin. In contrast, the length variation in IGS among *C*. *lentis* isolates is undoubtedly a relative recent event.

Considering that lentil cultivation in Western Canada remained disease free for decades [5] and that seeds for planting are produce locally, combined with the lack of other means of entry from the few countries where of *C*. *lentis* occasionally has been reported, it seems likely that the pathogen originated locally and made a host jump from an existing host species to lentil. The lentil pathogen in Western Canada could have originated from either flax (*Linum usitatissimum*) or bean (*Phaseolous vulgaris*) since *Colletotrichum* species from these crops also have falcate conidia, hemibiotroph infection process and high ITS homology [14, 7]. Alternatively, the pathogen could have originated from *Vicia* species, since both faba bean (V. *fabae*) and the common weed narrow-leaf vetch (*V*. *americana*) are highly susceptible to *C*. *lentis* from lentil [15]. A similar recent host-jump by another *Colletotrichum* sp. has been documented in coffee [16], and is one of several mechanisms by which new plant pathogens arise in agricultural crops [17]. Anthracnose became a serious problem for lentil producers in Manitoba in the late 1980’s [5], and subsequently spread to the neighbouring Province of Saskatchewan as well as North Dakota, USA [18, 13]. Dispersal was primarily by windblown lentil debris and dust generated during harvest carrying the pathogen’s resting structure in the form of microsclerotia [19].

To distinguish the two known races of *C*. *lentis* from lentil it had been necessary to inoculate differential lines with a spore suspension of individual single spore isolates. Since characterization of a large number of isolates requires a considerable amount of growth space and more than 30 days from planting to disease rating, we developed a PCR-based probe for discrimination of the races to replace the resource intensive pathogenicity test. After examining the IGS and ITS regions of ribosomal DNA and three genes commonly used in phylogenetic studies, a clear differentiation at the molecular level was identified in a minisatellite at the 3’-end of the IGS region consisting of a varying number of a perfect 39 nt repeat. A new probe, 39F/R, was designed around this length polymorphism which after PCR amplification and separation by gel electrophoresis showed one or two bands of 420 or 500 nucleotides (7 or 9 repeats) in race Ct1 isolates, while Ct0 isolates had other band sizes, primarily of 220 or 300 nucleotides (2 or 4 repeats), but never 420 or 500 nt. Despite a potential high sequence variation in a minisatellite of this size, in no case did this create an overlap between Ct0 and Ct1 isolates. We concurrently found that the 5S rDNA gene was absent from the IGS region in *C*. *lentis* and consequently must be dispersed throughout the genome. This is supported by the fact that the 5S gene in the newly sequenced genomes of both *C*. *higgisianum* and *C*. *graminicola* showed numerous hits to contigs on several chromosomes demonstrating that this gene is likewise located outside the IGS region in these related species [20].

When the 39F/R PCR probe was used to survey the frequency of repeat number in *C*. *lentis* isolates collected up to 20 years apart it was found that 2 and 4 repeats had increased in contrast to 7 and 9 repeats.

In the period between the two surveys several lentil varieties were developed with resistance to race Ct1 derived from the variety Indianhead [9, 10]. Currently, about one third of varieties have some resistance to this race [Saskatchewan Ministry of Agriculture 2014]. Only recently was lentil germplasm with resistance to race Ct0 identified, but these sources have not yet been utilized in lentil breeding [21].Since isolates with 2 and 4 repeats were consistent with race Ct0 in 1991–1999, it is plausible that the increase of isolates with these repeats in 2012 is related to a selection advantage of race Ct0 isolates due to the lack of Ct0 resistant lentil varieties.

A single 39 nt minisatellite was identified in 58% of isolates collected in 1991–1999 compared to 82% in 2012. The remainder of isolates had up to five minisatellites of different lengths mostly seen as minor bands in isolates from both surveys (Tables 1 and 4), confirming the well-known higher mutation rate permitted in the IGS region. Consequently, the frequency of some minisatellites in the population will be transient and indicates a fitness cost to isolates having multiple minisatellites.

The 39F/R PCR probe amplified the identical 39 nt repeat in all seven fungal species examined, including four *Colletotrichum* species from lentil, soybean, alfalfa and scentless chamomile as well as distantly related species, *A*. *lentis*, *S*. *sclerotiorum* and *B*. *cinerea*. Sequencing of DNA after separation of bands by gel electrophoresis confirmed the presence of a perfect 39 nt repeat in all cases regardless of band size and intensity. All isolates had between two and six minisatellites of different lengths ranging from 2 to 40 repeats. The probe was therefore not suitable as a diagnostic tool at the fungal species level. However, we confirmed that another PCR-based probe, G02FP/G02RP, previously published by Ford et al. [11], exclusively amplified a 1600 nt fragment in *C*. *lentis* from lentil, whereas no amplification products were found in any other fungal species. Accordingly, only when the *C*. *lentis* species is known can the 39F/R PCR probe be used to discriminate between isolates based on repeat number.

The source of minisatellite variation among individuals of the same organism includes nucleotide shifts, sequence length differences and copy number variation. Nucleotide shifts occur primarily by mutation, while length differences are foremost created by unequal crossing over of chromatids during mitosis and meiosis or slippage during transcription resulting in increase or decrease of repeat number [22, 23]. Several mechanisms can contribute to copy number variation such as transfer of genes, parts of chromosomes or entire chromosomes (supernumerary) which is particularly common among fungi [22, 23]. Notably, the 39 nt minisatellite lacked nucleotide shifts, while copy number varied from one to six and sequence length from 2 to 40 repeats both within and between fungal species (Table 5). Since the fungal species we examined represented various asexual and sexual lifestyles any of the mechanisms described above could have contributed to this variation. Copy number variation was not observed in the single spore cultures derived from *C*. *lentis* although they had undergone numerous mitotic events.

The fact that the highly conserved 39 nt minisatellite was present in all fungal pathogens shows its universal importance comparable to the rDNA genes themselves. Furthermore, the minisatellite was located just upstream of the 18S gene in a region known to harbour both transcription terminators and activators [24, 25, 26]. Moreover, the hairpin formed by each 39 nt repeat consisted of a GC rich stem plus a loop of five adenosine residues which is a typical site for stalling of RNA polymerase (Fig 3B). We also found that both the 16 nt pre-repeat and the following 39 nt repeat had high sequence homology with transcription termination factors (TTF) in other eukaryote species, 81% homology with *Saccharomyces carlbergensis* and 62% with human [27], all of which leads us to conclude that length polymorphisms in the 39 nt minisatellite may be associated with transcriptional regulation of the 18S gene as shown for human TTF-1 [28].

The highly variable 23 nt minisatellite in C. *lentis* was located downstream of the 26S gene, and consisted of ten diverse sequences organized in three patterns resulting in three length polymorphisms. This minisatellite also differentiated race Ct0 from Ct1 except for one isolate, 95H15. This near perfect relationship between race designation and repeat number, including the same order of slightly different repeat sequences, is notable considering that the number of such variations is theoretically extremely high in a minisatellite of this size. Genotypic variation in the IGS region indicates the isolate 95H15 could be different from race Ct1 and Ct0, but currently it cannot be identified due to the lack of suitable differential lentil lines.

High sequence homology was found both between the 23 nt and 39 nt repeats and between the 39 nt and the 16 nt pre-repeat (Fig 3A). It is therefore plausible that the 39 nt repeat, which is highly conserved among fungal species, gave rise to the more variable 23 nt repeat. Each repeat of the 23 nt minisatellite consisted of 10 conserved and 13 variable nucleotides. Interestingly, uracil was at the first position in all RNA-translated sequences (Table 2). Uracil at the 5’-end is a common feature of small RNA (sRNA) as shown in transcriptomes from several fungi including *Neurospora crassa* [29], *Magnaporthe oryzae* [30], *S*. *sclerotiorum* [31] and *B*. *cinerea* [32]. The majority of small RNA from *B*. *cinerea* also have U in the first position, which favour binding to the Argonaut protein in the host plant, and is the first step in hijacking of the immune system as described by Weiberg et al. [32]. Intriguing studies demonstrate ways in which sRNA are involved in host-pathogen interaction, one in which sRNA in *B*. *cinerea* bind and silence defense genes in *Arabidopsis thaliana* to establish infection [32], and another where sRNA in *Phytophthora sojae* silence its own virulence genes thereby avoiding detection by resistance genes in soybean (*Glycine max*) [33]. In fact, the non-coding sequences in rDNA and the nucleolus in general are emerging as important locations for post transcriptional gene regulation in response to different biotic and abiotic stress factors [34, 35]. There is also evidence directly linking synthesis of a certain group of sRNA to ribosomal DNA [29]. Taken together we hypothesize that the 23 nt minisatellite in *C*. *lentis* could be a site of sRNA synthesis, that by one of the cited mechanisms could contribute to different pathogenicity of the two races, Ct0 and Ct1. Lentil accessions resistant to one or both races exist [8, 21] and resistance genes against race Ct1 have been identified likely governed by the well known gene-for-gene interaction [36]. How fungal small RNA affects this interaction needs further investigation and is best carried out in a well characterized host-pathogen system amendable to gene recombination and genetic manipulation of one or both organisms. The lentil—*C*. *lentis* interaction is still in its infancy, and although crossing of *C*. *lentils* isolates is possible *in-vitro* it is not routine and the sexual stage has not been found in nature [37]. Evidence supporting our hypothesis that IGS is a site of small RNA synthesis affecting pathogenicity should therefore be sought in other host-pathogen systems. Good candidates are those where sequence length polymorphism and repeat variation in the IGS region have already been used to differentiate closely related species such as *Alternaria* [38] and *Fusarium* [39, 40]. Isolates have been grouped with the help of IGS polymorphisms such as in *Pyrenophora graminea* [41, 42], *S*. *sclerotiorum* [43], *Verticillium dahlia* [44], *Diaporthe helianthi* [45], *Fusarium avenaceum* [46], *F*. *monoliforme* [47], and different forma speciales of *F*. *oxysporum* [48–52]. A few reports exist in which IGS variation was related to host range as demonstrated in *Puccinia* sp. causing leaf and stem rust in cereals [53, 54]. Here species specific variation in IGS differentiated *P*. *graminis* f. sp. *tritici*, *P*. f. sp. *secalis*, *P*. f. sp. *avenae*, *P*. *recondita* f. sp. *tritici* and *P*. *coronate* f. sp. *avenae*, most notably some IGS polymorphisms were related to races within *P*. *graminis* f. sp. *tritici*. These studies combined with our identification of IGS minisatellites as potential sites of RNA polymerase regulation and small RNA synthesis affecting host range and pathogenicity of races warrant further investigation.

## Materials and Methods

### Ethics statement

No specific permits were required for the described field studies. This work did not involve endangered or protected species.

### Fungal isolates

A total of 50 *C*. *lentis* isolates from lentil representative of two races were selected for development of a PCR-based probe. The fungal isolates were obtained from anthracnose infected lentil plants and seed samples collected in the Canadian Provinces of Saskatchewan and Manitoba between 1991 and 1999 (Table 1). These samples were part of annual disease surveys published in the Canadian Plant Disease Survey, thus specific permission for sampling of isolates was not required.

Isolates were grown on half strength oat meal agar (½ OMA, Difco) in 9 cm Petri plates and incubated in a 22°C/18°C day/night cycle with 16 h light. Single spore cultures were made from each isolate by flooding a culture with 1 ml sterile water and streaking 10 μl spore suspension onto 2.0% water agar. After 1–2 days incubation under the same conditions as just described, single germinating spores were collected under a light microscope and transferred to fresh ½ OMA plates. Plugs with mycelium and spores were taken from actively growing cultures and stored in cryo-freezer solution (10% skim milk and 40% glycerol) at -80°C until needed for DNA extraction. An additional set of 9 to 20 single spore isolates were generated from three Ct0 isolates, and one Ct1 isolate repeating the single spore method just described (Table 1).

The 50 *C*. *lentis* isolates were determined to belong to either race Ct0 or Ct1 by inoculating differential lentil lines as described by Buchwaldt et al. [8]. Race designation was previously determined for 28 of these isolates [8], and data was obtained for an additional 22 isolates using the differential lines, Indianhead and PI320937 [USDA—Agriculture Research Service, Pullman, WA, USA] resistant to race Ct1 isolates but susceptible to race Ct0, and cultivar Eston which is susceptible to all isolates. The pathogenicity test entailed inoculation of plants at the early flowering stage with a spore suspension (10^−5^ spores ml^-1^ water, 1.5 ml per plant) followed by incubation at 100% relative humidity for 24 h. Susceptible lines developed typical anthracnose symptoms 10 to 14 days after inoculation consisting of deep penetrating necrotic lesions on stems and severe leaf lesions causing defoliation. In contrast, resistant lines had no symptoms or only a few superficial stem and leaf lesions [8].

Selected isolates representative of race Ct0 and Ct1 were used for sequencing of three gene fragments in the translation elongation factor 1α (*tef1α*), RNA polymerase II subunit B2 (*rpb2*), and ATP citrate lyase subunit A (*acla*), as well as the intergenic spacer (IGS) and internal transcribed spacer (ITS) regions of ribosomal DNA (Table 1).

A total of 219 isolations of *C*. *lentis* were made in 2012 from anthracnose infected lentil seed samples provided by the seed testing company in Discovery Seed Labs. Samples were identified by town and subsequently given a number from 1 to 4. The owners identity was not provided, thus no specific permission was required.

These infected samples were initially identified by the seed testing company Discovery Seeds (Saskatoon, Saskatchewan, Canada) and originated from 74 commercial lentil fields in Saskatchewan. The presence of *C*. *lentis* had been determined by plating surface sterilized seeds on nutrient agar (200 seeds per field). Subsequently, we made isolations of *C*. *lentis* from one to four infected seeds from each location. All isolates were grown on ½ OMA and stored at -80°C as described above until needed for DNA extraction.

*Colletotrichum* isolates from host species other than lentil were included in the study: one *C*. *truncatum* isolate developed as a biocontrol agent for scentless camomile (*Matricaria perforata* Mérat) [55], three *C*. *trifolii* isolates from alfalfa (*Medicago sativa* L) and three *C*. *gloeosporioides* (Penz.) Penz and Sacc isolates from soybean (*Glycine max* L) (Table 5). These isolates were grown on ½ OMA and stored at -80°C until needed for DNA extraction. Other fungal pathogens commonly found on lentil were also examined, five isolates of *Ascochyta lentis* Vassiljevsky and three each of *Botrytis cinerea* Pers. Fr. and *Sclerotinia sclerotiorum* Lib. De Bary (Table 5). These isolates were grown on potato dextrose agar (PDA) and stored at -80°C until needed for DNA extraction.

### DNA extraction

Isolates stored at -80°C were transferred to ½ OMA for *Colletotrichum* and to PDA for the other fungal species. After 7–10 days incubation under conditions described above each culture was flooded with 20 ml sterile water for 1 h. The surface was scraped with a sterile glass slide to dislodge spores and mycelium with minimal disturbance of the agar surface. The suspension was collected in a 50 ml sterile Falcon tube and incubated at room temperature overnight to soften the cell walls. For DNA extraction the suspension was centrifuged at 3000 rpm for 5 min (Sorvall RT7 Benchtop Centrifuge, GMI, Ramsey, MN, USA) and the supernatant discharged. The resulting fungal pellet was resuspended in 1 ml water, transferred to a 1.5 ml Eppendorf tube, and centrifuged at 14.000 rpm for 2 min (Eppendorf 5430, Hamburg, Germany). After removing the supernatant the pellet was resuspended in 500 μl 2xCTAB buffer (2% CTAB, 20 mM EDTA, 100 mM Tris-HCl pH 8.0, 1.4 M NaCl, 0.2% 2-mercaptoethanol), ground with a sterile pestle and incubated in 0.5 ml 24:1 chloroform-isoamyl alcohol at 65°C for 1.5 h, after which the samples were vortexed for 30 sec (Vortex Genie, Scientific Industries, Bohemia, NY, USA). The sample was again centrifuged at 14.000 rpm for 10 min and the supernatant transferred to a new Eppendorf tube and 0.6 volumes of isopropanol added. After mixing by gentle inversion the suspension was placed at room temperature in the dark to precipitate the DNA. The sample was centrifuged for 20 min at 14.000 rpm, washed with 70% ethanol and centrifuged for an additional 10 min. The resulting DNA pellet was air dried and resuspended in 1xTE with 0.2 μg μl^-1^ RNAse A (Qiagen, Hilden, Germany). The DNA was quantified on a Nanodrop 1000 v3.7 (Thermo Fisher Scientific, Waltham, MA, USA) and diluted to 10–40 ng μl^-1^ with 1xTE.

### Analysis of *tef1α*

The 5’ end of *tef1α* was amplified and sequenced in seven Ct0 isolates and six Ct1 isolates (Table 1). DNA was amplified using degenerate primers, tef71f and tef997r, and sequencing was carried out with tef85f and tef954r [13] (S1 Table). The PCR amplification reaction consisted of 1 μl fungal DNA template (10–40 ng μl^-1^), 0.32 μl 5 μM of each primer tef71f and tef997r with puRe Taq Ready-To-Go PCR Beads (GE Healthcare, Piscataway, NJ, USA) and water for 25 μl final volume. PCR touchdown conditions were initial denaturation at 94°C for 4 min, followed by 4 cycles each at 94°C for 60 sec and 70°C for 90 sec, then 72°C for 90 sec, followed by 26 cycles with the annealing temperature reduced by 0.5°C per cycle from 68°C to 55°C, 12 cycles with annealing kept at 55°C, and a final elongation at 72°C for 10 min (GeneAmp PCR System 2700, Life Technologies, Waltham, MA, USA). Each resulting DNA amplicon was sequenced in reactions containing 1.0 μl PCR-derived DNA template (10–40 ng μl^-1^), 0.4 μl 5 μM of either primer tef85f or tef954r, 1.0 μl BigDye (Life Technologies, Waltham, MA, USA), 1.0 μl halfBD (BioCan Scientific, Mississauga, ON, Canada) and 1.6 μl water for 5 μl final volume. The sequencing conditions were 25 cycles each at 96°C for 30 sec and 58°C for 15 sec, then 60°C for 4 min (ABI Prism 3100 Genetic Analyzer, Applied Biosystems, Life Technologies, Waltham, MA, USA). The resulting DNA sequences were visualized with Sequencher 5.0 (Gene Codes Corporation, Ann Arbor, MI, USA) and aligned with Clustal X software [56].

### Analysis of *rpb2*

A fragment of *rpb2* was amplified and sequenced in three isolates of race Ct0 and Ct1, respectively (Table 1). DNA was amplified using degenerate primer pair RPB2-210up and RPB2-1450low, and sequencing was carried out with PB2-210up and RPB2-1150low (S1 Table). The PCR amplification reaction consisted of 1 μl fungal DNA template (10–40 ng μl^-1^), 0.32 μl 5 μM of each primer RPB2-210up and RPB2-1450low with puRe Taq Ready-To-Go PCR Beads and water for 25 μl final volume. The PCR touchdown conditions were initial denaturation at 94°C for 3 min, 5 cycles each at 94°C for 45 sec and 60°C for 45 sec, then 72°C for 2 min, followed by 5 cycles with the annealing temperature reduced by 1.0°C per cycle from 58°C to 54°C, then 30 cycles with annealing kept at 54°C and final elongation at 72°C for 10 min. Each resulting DNA amplicon was sequenced in reactions containing 1.0 μL PCR-derived DNA template (10–40 ng μl^-1^), 0.4 μL 5 μM of either primer PB2-210up or RPB2-1150low, 1.0 μL BigDye, 1.0 μL halfBD and 1.6 μL water for 5 μl final volume. The sequencing conditions were 40 cycles each at 96°C for 30 sec and 50°C for 15 sec, then 60°C for 3 min. The resulting DNA sequences were visualized with Sequencher 5.0 and aligned with Clustal X software.

### Analysis of *acla*

A fragment of the large subunit of ATP citrate lyase (*acla*) was amplified and sequenced in three isolates each of race Ct0 and Ct1 (Table 1). DNA was amplified and sequenced using primers acl1-230up and acl1-1220low [Gräfenhan laboratory] (S1 Table). The PCR amplification reaction consisted of 1 μl fungal DNA template (10–40 ng μl^-1^), 0.32 μl 5 μM of each primer acl1-230up and acl1-1220low with puRe Taq Ready-To-Go PCR Beads and water for 25 μl final volume. The PCR conditions were initial denaturation at 95°C for 3 min, 5 cycles each at 95°C for 45 sec and 60°C for 45 sec, then 72°C for 2 min, followed by 5 cycles with annealing at 58°C, then 30 cycles with annealing at 56°C, and final elongation at 72°C for 8 min. Each resulting DNA amplicon was sequenced in reactions containing 1.0 μL (10–40 ng) PCR-derived DNA template, 0.4 μL 5 μM of either primer acl1-230up or acl1-1220low, 1.0 μL BigDye, 1.0 μL halfBD, 1.6 μL water for 5 μl final volume. The sequencing conditions were 95°C for 3 min, followed by 40 cycles each at 95°C for 30 sec and 50°C for 15 sec, then 60°C for 2 min. The resulting DNA sequences were visualized with Sequencher 5.0 and aligned with Clustal X software.

### Analysis of ITS

The complete ITS1- ITS2 region was amplified and sequenced in four isolates of race Ct0 and six of race Ct1 (Table 1). The generic primer pair, ITS4 and ITS5, [57] was used for both PCR amplification and sequencing (Fig 1 and S1 Table). The PCR amplification reaction contained 1.0 μl fungal DNA template (10–40 ng ul^-1^), 0.32 μl 5 μM each of primer ITS4 and ITS5 with puRe Taq Ready-To-Go PCR Beads and water for 25 μl final volume. The PCR conditions were initial denaturation at 94°C for 3 min, followed by 40 cycles each at 94°C for 60 sec and 58°C for 75 sec, then 72°C for 105 sec, and final elongation at 72°C for 10 min. The PCR amplification product was purified on a spin column as per manufacturer’s instructions (UltraClean PCR Clean-up, MoBio Laboratories). Each resulting DNA amplicon was sequenced in reactions containing 1.0 μl PCR-derived DNA template (10–40 ng ul^-1^), 0.4 μl 5 μM of either primer ITS4 or ITS5, 1.0 μl BigDye, 1.0 μl halfBD and 1.6 μl water for 5 μl final volume. The sequencing conditions were 25 cycles each at 96°C for 30 sec and 58°C for 15 sec, then final elongation at 60°C for 4 min (ABI Prism 3100 Genetic Analyzer, Applied Biosystems, Life Technologies). The DNA sequences were visualized with Sequencher 5.0 and aligned with Clustal X software.

### Analysis of IGS

In preparation for sequencing of the complete IGS region in *C*. *lentis* from lentil the generic primer pair LR12R and invSR1R [Vilgalys’ laboratory] (S1 Table) was used to first amplify the region from the ends of the 26S and 18S genes (Fig 1) in four isolates of race Ct0 and three isolates of race Ct1 (Table 1). The PCR amplification reaction consisted of 1.0 μl fungal DNA template (10–40 ng μl^-1^), 0.32 μl 5 μM of each primer LR12R and invSR1R, puRe Taq Ready-To-Go PCR beads, and water for 25 μl final volume. The PCR conditions were initial denaturation at 94°C for 3 min, followed by 35 cycles each at 95°C for 60 sec and 55°C for 90 sec, then 72°C for 5 min, and final elongation at 72°C for 10 min. The amplification product was purified on a spin column as per manufacturer’s instruction (UltraClean PCR Clean-up, MoBio Laboratories). Each resulting DNA amplicon was sequenced in reactions containing 1.0 μl PCR-derived DNA template (10–40 ng μl^-1^), 0.4 μl 5 μM of either primer LR12R or invSR1R, 1.0 μl BigDye, 1.0 μl halfBD, 1.6 μl water with 1/8 dilution of halfBD for 5 μl final volume. Sequencing conditions were 25 cycles each at 96°C for 30 seconds and 50°C for 15 seconds, followed by 60°C for 4 min. DNA sequences were visualized with Sequencher 5.0 and aligned with Clustal X software. Based on these initial IGS sequences we designed four new primers, CtGSF336, CtGSF970, CtGSR1704 and CtGSR2266 (S1 Table) at about 700 nt intervals for further sequencing and assembly of the complete IGS region (Fig 1). Sequencing was performed with eight isolates of race Ct0 and eight of race Ct1 (Table 1) with the same reaction and run conditions as described for the two generic primers. The complete IGS region was assembled for each isolate with MAFFT v.7 with the E-INS-i strategy for long gaps [58].

### Design of 39F/R PCR probe

The complete IGS sequence from sixteen *C*. *lentis* isolates were aligned for identification of polymorphisms distinguishing the two races. The most significant polymorphism was a minisatellite comprising a varying number of a 39 nucleotide perfect repeat, CCAAGTACCCTGGAAAAAGTCCCAGGTACCCTGGAAAGG (Fig 4). A new primer pair, 39F and 39R, was designed for amplification of this length polymorphism specifically (S1 Table). The forward primer, 39F (5’-GAG ATA AGT AAA GAC GGA GAT AAA-3’), was designed to anneal 123 nt upstream of the minisatellite to ensure appropriate sized amplicons for subsequent separation by gel electrophoresis. The reverse primer, 39R (5’-TAG GCG CCA AGG TAG AAA GT-3’), was designed downstream one nucleotide away from the last 39 nt repeat (Fig 4).

### Analysis of the 39 nt minisatellite in *C*. *lentis*

The 39 nt minisatellite was amplified using the 39F/R PCR probe in 50 *C*. *lentis* isolates collected between 1991 and 1999 (Table 1). The PCR amplification reaction consisted of 1.0 μl fungal DNA template (10 ng μl^-1^), 1.0 μl 0.5 μM of each primer 39F and 39R, 5 μl FideliTaq PCR Master Mix (Affymetrix, Santa Clara, CA) in buffer with a final concentration of 0.05 mM dNTPs, 0.5 mM MgCl,12.5 mM KCl and 2.5 mM Tris-HCl (pH 8.3) and water for 20 μl final volume. The PCR conditions were optimized and consisted of initial denaturation at 94°C for 30 sec, followed by 35 cycles each at 94°C for 30 sec and 52°C for 30 sec, then 68°C for 90 sec, and final elongation at 68°C for 5 min (PTC-200 Thermo Cycler, MJ Research, Waltham, MA).

Amplification products were separated by gel electrophoresis in 1.5% agarose gels. Wells were loaded with 20 μl PCR-derived fungal DNA template plus 4 μl dye (0.25% v/v bromophenol blue, 15% Ficoll PM 400, Sigma, St. Louis, MO, USA). Lanes with ladders were loaded with 15 μl 10 ng μl^-1^ 1 KB Plus DNA Ladder (Invitrogen Life Technologies, Carlsbad, CA). Gels were run at 90 V for 2.5 hr (Sub-Cell GT Agarose Gel System, BioRad, Hercules, CA, USA), stained with GelRed as per manufactures instructions (Biotium, Hayward, CA, USA), and bands visualized under UV light. Standardization of all steps allowed visual discrimination of major and minor bands based on their relative size and brightness.

A total of 25 bands from agarose gels were selected for DNA sequencing representative of band sizes 220, 300, 380, 450 and 600 nt in twelve Ct0 isolates, and 420 and 500 nt in nine Ct1 isolates (Table 1). These bands were excised and DNA extracted as per manufacturer’s instructions (QIAquick Gel Extraction Kit, Qiagen, Hilden Germany). The DNA sequencing reaction was 2.0 μl PCR-derived DNA template (20 ng μl^-1^), 1.8 μl 3.2 μM of each primers 39F and 39R, 1.0 μl BigDye, and 5.2 μl water for 10 μl final volume. The PCR conditions were initial denaturation at 96°C for 60 sec, 27 cycles each at 96°C for 15 sec and 50°C for 6 sec, then final elongation at 60°C for 4 min (3730xl DNA Analyzer, Applied Biosystems, Life Technologies, Waltham, MA, USA). The DNA sequences were aligned with Sequencher 5.0 software. Sequencing results showed identical 39 nt repeats in all excised bands regardless of size and brightness. Thus, the number of 39 nt repeats in each band was calculated as (band size—144 nt) divided by 39 nt; where 144 nt comprise 66 nt with the annealing site of the forward primer (39F), the 16 nt and 41 nt pre-repeats plus 21 nt with the annealing site of the reverse primer (39R) (Fig 4). For band sizes above 750 nt, which fell outside of the resolving power of gel electrophoresis, the repeat number was estimated, i.e. for 15 repeats or higher.

### Analysis of IGS sequence similarities

The complete IGS sequence from *C*. *lentis* race Ct0 isolate 95SP31 was compared against itself using LALIGN software to obtain alignment and similarity scores, while PLALIGN software was used to produce a dot-plot graph [FASTA version 36.3][59].

### Analysis of DNA secondary structure

Secondary structure predictions and calculation of free energy values of the 23 and 39 nt minisatellites were performed using the Mfold DNA folding server [60] with the provided default free-energy parameters. Analysis of the 39 nt minisatellite were performed with a stepwise increase from two to twelve of the repeats including 123 nt upstream and 21 nt downstream of the minisatellite in all cases (Fig 4). Analysis of the 23 nt minisatellite were performed for three sequence lengths with 14, 17 and 19 repeats, respectively (Table 3).

### Analysis of the 39 nt minisatellite in other fungal species

The presence of the 39 nt minisatellite was examined in other fungal species. PCR amplification using the 39F/R probe was undertaken with DNA from one *C*. *truncatum* isolate (scentless camomile), three *C*. *trifolii* isolates (alfalfa), three *C*. *gloeosporioides* isolates (soybean), five *Ascochyta lentis* isolates (lentil), three *Botrytis cinerea* isolates (lentil) and three *Sclerotinia sclerotiorum* isolates (lentil), with six *C*. *lentis* isolates from lentil included for comparison (Table 5). PCR conditions, gel electrophoresis and visualization of bands were the same as described above for analysis of the 39 nt minisatellite in *C*. *lentis*. Standardization of all steps allowed visual discrimination of major and minor bands based on their relative size and brightness (S3 Fig). A total of 10 bands from agarose gels were selected for DNA sequencing representative of band sizes 220, 300, 340 and 450 nt (Table 5). DNA extraction, amplification and sequencing were the same as described above for analysis of the 39 nt minisatellite.

### Analysis of G02FP/G02RP PCR probe

We tested a previously published PCR-based primer pair, G02FP and G02RP, shown to amplify a 1600 nt product in an unknown region of the *C*. *lentis* genome specific to this pathogen from lentil [11]. We examined the specificity of this primer using both races of *C*. *lentis* from lentil, *C*. *truncatum* from scentless chamomile, *C*. *gloeosporioides* from soybean, and *C*. *trifolii* from alfalfa, and fungal pathogens isolated from lentil including *A*. *lentis*, *B*. *cinerea* and *S*. *sclerotiorum*. The original DNA amplification method by Ford et al. [11] was optimized for use with FideliTaq polymerase. The PCR amplification reaction contained 1.0 μl fungal DNA template (10 ng μl^-1^), 1.0 μl each of 0.5 μM G02FP and G02RP, 5 μl FideliTaq PCR Master Mix, 5 μl of buffer, 0.05 mM, dNTPs, 0.5 mM MgCl, 12.5 mM KCl, and 2.5 mM Tris-HCl (pH 8.3) and water for 20 μl final volume. The PCR conditions were initial denaturation at 94°C for 2 min, followed by 35 cycles each at 94°C for 30 sec and 62°C for 30 sec, then 68°C for 90 sec, and final elongation at 68°C for 5 min. Amplification products were separated by gel electrophoresis, stained with GelRed and visualized under UV light as described above for analysis of the 39 nt minisatellite in *C*. *lentis*.

## Supporting Information

S1 Fig Agarose gel of amplification products from *Colletotrichum lentis* race Ct0 and Ct isolates using the 39F/R PCR probe. Isolates belonging to race Ct0 had 2 or 4 repeats (lane 11 to 18), while isolates belonging to race Ct1 had 7 or 9 repeats (lane 2 to 9). The number of repeats was calculated as (band size– 144) / 39.
(TIF)

S2 Fig Agarose gel of amplification products from *Colletotrichum lentis* single spore isolates using the 39F/R PCR probe. Five single spore cultures from isolate May9918 showed identical banding pattern of 5 and 6 repeats (lane 2 to 6). Similarly, nine single spore cultures from isolate 95N2 showed an identical band of 7 repeats (lane 8 to 16).
(TIF)

S3 Fig Agarose gel of amplification products from diverse fungal species using the 39F/R PCR probe. An identical minisatellite containing 39 nt repeats was identified in all fungal species examined. The number of repeats from 2 to 40 repeats was calculated as (band size– 144) / 39.
(TIF)

S1 Table Primers used for DNA amplification and sequencing in the present study. IGS (intergenic spacer), ITS (internal transcribed spacer), tef1α (translation elongation factor 1α), rpb2 (RNA polymerase II subunit B2), acla (ATP citrate lyase subunit A) and one unknown genomic region.
(DOCX)

S2 Table Free energy of secondary structures in two minisatellites from the intergeneic spacer region of *Colletotrichum lentis*. (DOCX)

S3 Table Number of a 39 nt repeat amplified by PCR probe 39F/R in IGS of 219 *Colletotrichum lentis* isolates from lentil seed samples in 2012. Amplification products were separated by gel electrophoresis and visualized by GelRed staining. The number of repeats was calculated as (band size -144 /39)
(XLSX)
