# Supplementary figures and images for: IGS Minisatellites Useful for Race Differentiation in Colletotrichum lentis and a Likely Site of Small RNA Synthesis Affecting Pathogenicity
Source: PLoS One. 2015 Sep 4;10(9):e0137398. doi: 10.1371/journal.pone.0137398 (PMC4560493; doi:10.1371/journal.pone.0137398)

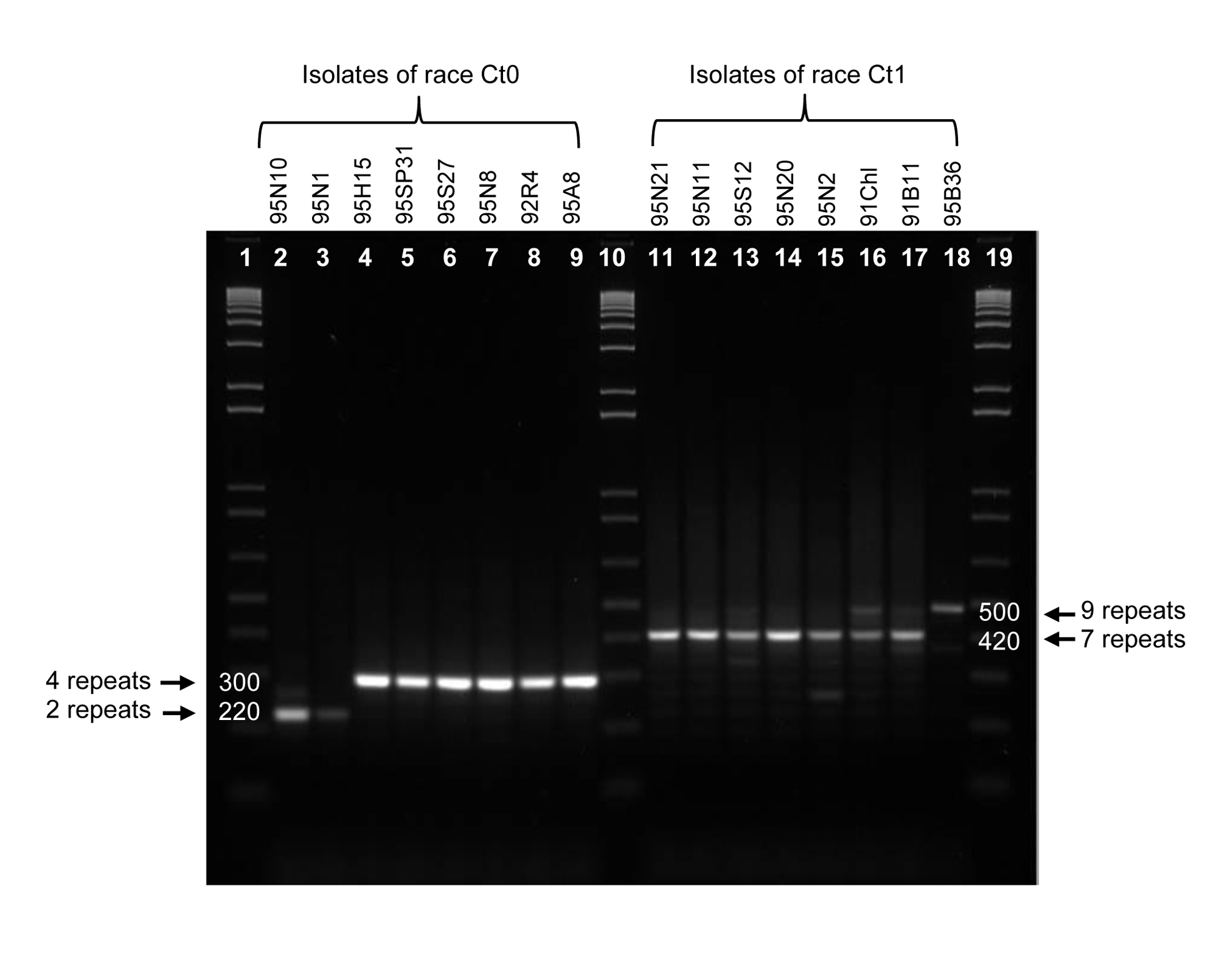

Supplement: S1 Fig — Isolates belonging to race Ct0 had 2 or 4 repeats (lane 11 to 18), while isolates belonging to race Ct1 had 7 or 9 repeats (lane 2 to 9). The number of repeats was calculated as (band size– 144) / 39. (TIF) [file pone.0137398.s001.tif]

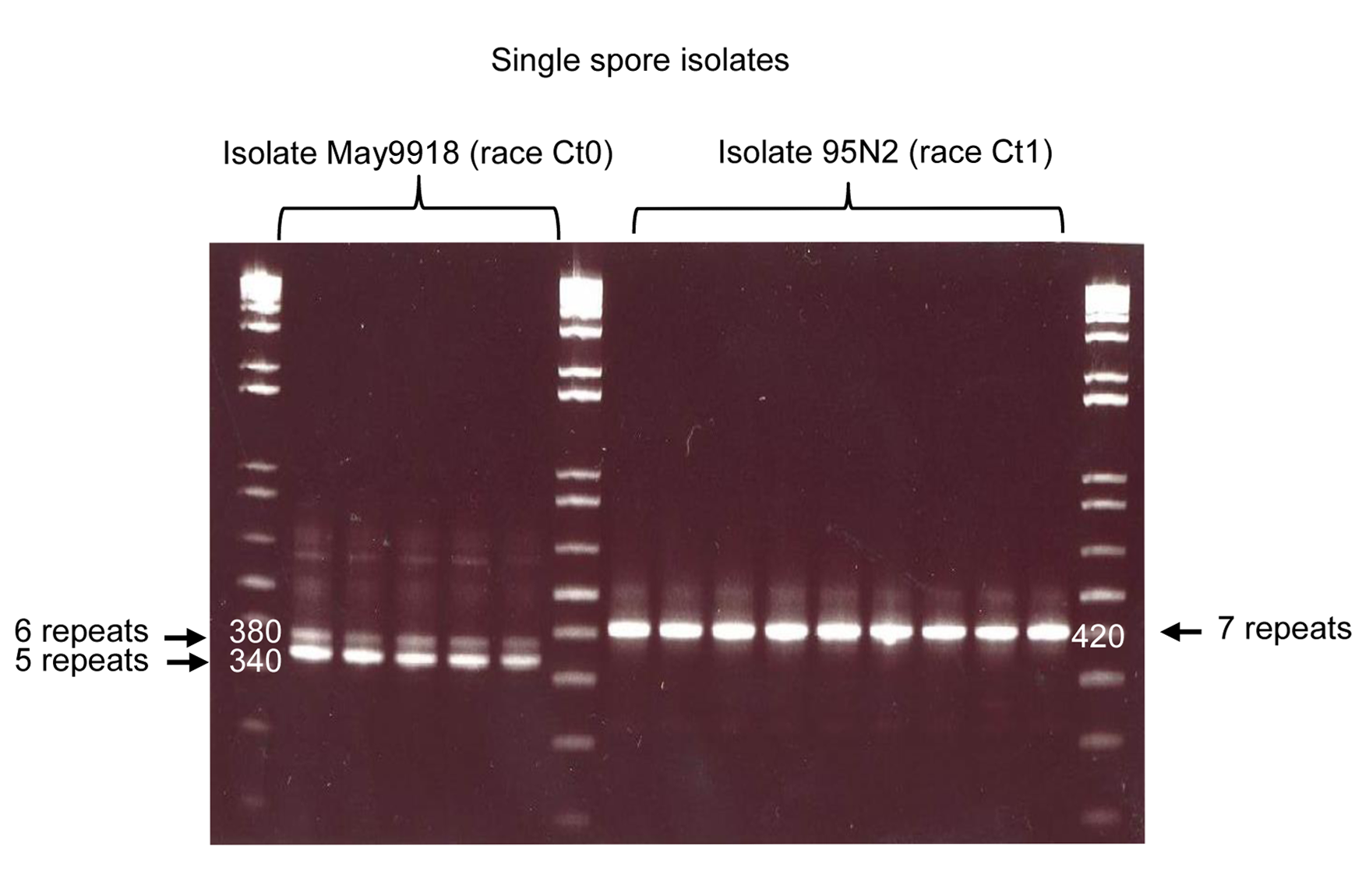

Supplement: S2 Fig — Five single spore cultures from isolate May9918 showed identical banding pattern of 5 and 6 repeats (lane 2 to 6). Similarly, nine single spore cultures from isolate 95N2 showed an identical band of 7 repeats (lane 8 to 16). (TIF) [file pone.0137398.s002.tif]

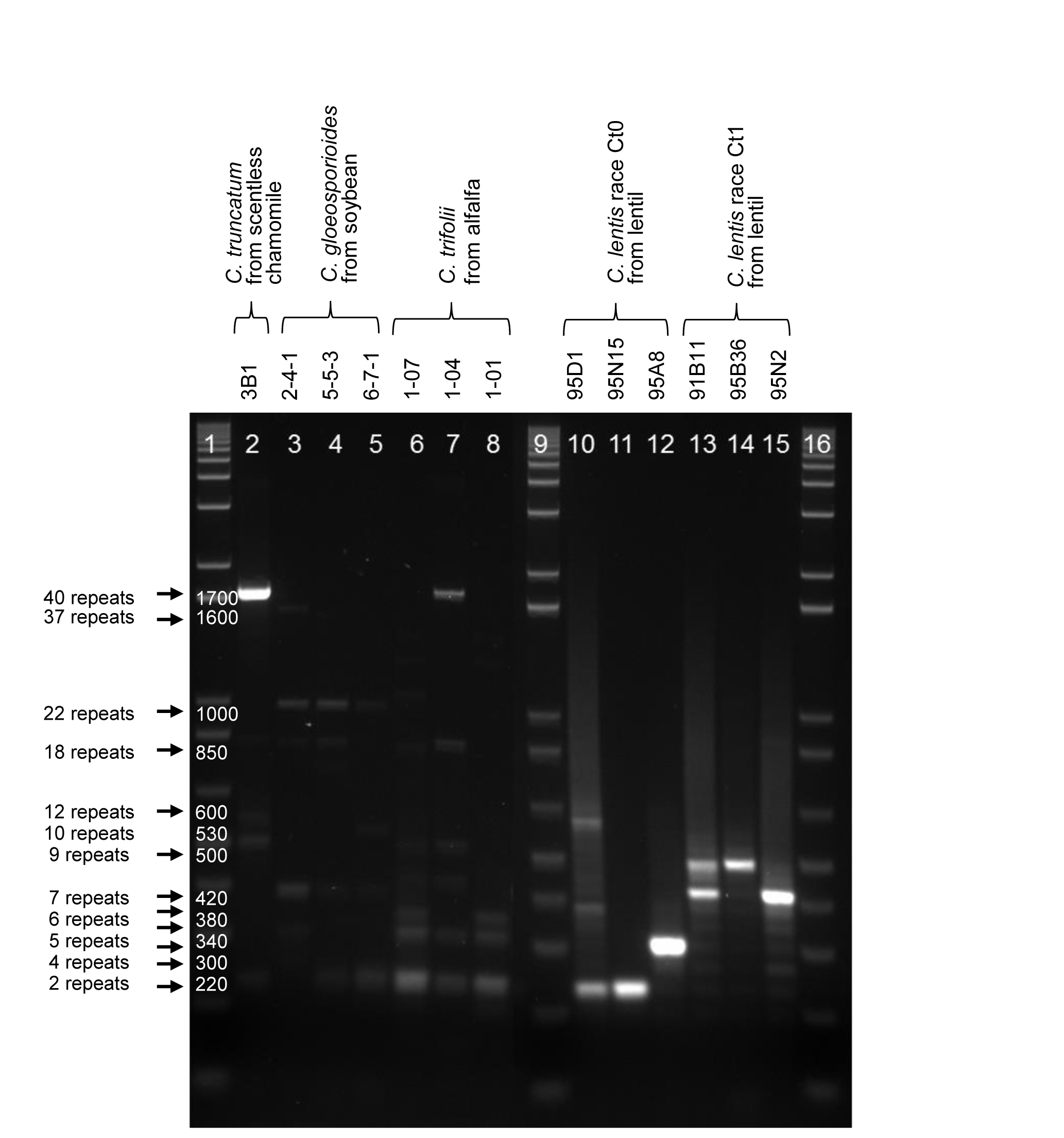

Supplement: S3 Fig — An identical minisatellite containing 39 nt repeats was identified in all fungal species examined. The number of repeats from 2 to 40 repeats was calculated as (band size– 144) / 39. (TIF) [file pone.0137398.s003.tif]
