# Supplementary material for: IGS Minisatellites Useful for Race Differentiation in Colletotrichum lentis and a Likely Site of Small RNA Synthesis Affecting Pathogenicity
Source: PLoS One. 2015 Sep 4;10(9):e0137398. doi: 10.1371/journal.pone.0137398 (PMC4560493; doi:10.1371/journal.pone.0137398)
Supplement: S1 Table — IGS (intergenic spacer), ITS (internal transcribed spacer), tef1α (translation elongation factor 1α), rpb2 (RNA polymerase II subunit B2), acla (ATP citrate lyase subunit A) and one unknown genomic region. (DOCX) [file pone.0137398.s004.docx]

| Amplified DNA region | Primer name | Orientation | Nucleotide sequence in 5’ to 3’ orientation | Reference |
| --- | --- | --- | --- | --- |
| *tef1α* | tef71f# | Forward | CAA AAT GGG TAA GGA GGA SAA GAC | Hoyos-Carvajal et al. 2009 |
|  | tef997r# | Reverse | CAG TAC CGG CRG CRA TRA TSA G |  |
|  | tef85f | Forward | AGG ACA AGA CTC ACA TCA ACG |  |
|  | tef954r | Reverse | AGT ACC AGT GAT CAT GTT CTT G |  |
| *rpb2* | RPB2-210up# | Forward | TGG GGW GAY CAR AAR AAG G | Gräfenhan's laboratory |
|  | RPB2-1450low# | Reverse | CAT RAT GAC SGA ATC TTC CTG GT |  |
|  | RPB2-1150low# | Reverse | GGT TGT GAT CRG GRA ARG GAA TG |  |
| *acla* | acl1-230up | Forward | AGC CCG ATC AGC TCA TCA AG | Gräfenhan et al. 2011 |
|  | acl1-1220low# | Reverse | CCT GGC AGC AAG ATC VAG GAA GT |  |
| ITS | ITS4 | Forward | TCC TCC GCT ATT GAT ATG C | White et al. 1990 |
|  | ITS5 | Reverse | GGA AGT AAA AGT CGT AAC AAG G |  |
| IGS from 5' end in 26S | LR12R | Forward | GAA CGC CTC TAA GTC AAT CC | Vilgalys' laboratory |
| IGS from 3' end in 18S | invSR1R | Reverse | ACT GGC AGA ATC AAC CAG CTA |  |
| Four regions within IGS | CtIGSF336 | Reverse | GTC GTA GGT AGA GGA AAA GG | Present study |
|  | CtIGSF960 | Forward | GAT TAG GAT GAT GAA GGA GAT GAT G |  |
|  | CtIGSR1704 | Reverse | CAT CCA TCA TAT CCC GAC ATC T |  |
|  | CtIGSR2266 | Reverse | GGC TAC TCT TCT CTC GCT CAC GT |  |
| 39 nt repeat within IGS | 39F | Forward | GAG ATA AGT AAA GAC GGA GAT AAA |  |
|  | 39R | Reverse | TAG GCG CCA AGG TAG AAA GT |  |
| Unknown | G02FP | Forward | GGC ACT GAG GAC GTC CTG AC | Ford et al. 2004 |
|  | G02RP | Reverse | GGC ACT GAG GTA AGC GCT TAG G |  |

**Table S1** Primers used for DNA amplification and sequencing in the present study. IGS (Intergenic Spacer), ITS (Internal 856 Transcribed Spacer), tef1α (translation elongation factor 1α), rpb2 (RNA polymerase II subunit B2), acla (ATP citrate lyase subunit A) and one unknown genomic region.

# Degenerate primers.
