# Supplementary material for: IGS Minisatellites Useful for Race Differentiation in Colletotrichum lentis and a Likely Site of Small RNA Synthesis Affecting Pathogenicity
Source: PLoS One. 2015 Sep 4;10(9):e0137398. doi: 10.1371/journal.pone.0137398 (PMC4560493; doi:10.1371/journal.pone.0137398)
Supplement: S2 Table — (DOCX) [file pone.0137398.s005.docx]

| Minisatellite | Number of repeats | Delta G kcal mol^-1^ |
| --- | --- | --- |
| 39 nt minisatellite | 2 | -25.1 |
|  | 3 | -32.1 |
|  | 4 | -39.1 |
|  | 5 | -46.0 |
|  | 6 | -53.0 |
|  | 7 | -60.0 |
|  | 8 | -67.0 |
|  | 9 | -73.9 |
|  | 10 | -80.9 |
|  | 11 | 87.9 |
|  | 12 | -94.8 |
| 23 nt minisatellite | 14 | -42.3 |
|  | 17 | -49.8 |
|  | 19 | -55.6 |

**Table S2** Free energy of secondary structures in two minisatellites from the intergeneic spacer region of *Colletotrichum lentis*.
